# Gender differences in students’ disaster knowledge and needs: A case study from Klaten, Indonesia

**DOI:** 10.4102/jamba.v17i1.1883

**Published:** 2025-08-29

**Authors:** Ratih P. Dewi, Wahyu Widiyatmoko, Yunus A. Wibowo, Puspita I. Wardhani, Muhamad T. Hidayat

**Affiliations:** 1 Department of Geography Education, Faculty of Teacher Training and Education, University of Muhammadiyah Surakarta, Surukarta, Indonesia; 2 School of Education, Culture and Society, Faculty of Education, Monash University, Clayton, Australia

**Keywords:** disaster needs, earthquake, disaster education policy, gender, knowledge

## Abstract

Knowledge is one of the critical aspects of disaster risk reduction capacity. Attributes that exist in an individual, such as age and gender, can be a factor in their high or low knowledge. This study aims to determine (1) the difference in students’ knowledge about earthquake disasters based on gender and (2) the difference in student needs in gender-based learning. This study is quantitative research with a comparative causal design. This research was conducted in four schools: Public Junior High School Jabung, Public Junior High School 1 Bayat, Private Vocational High School Berbudi Gantiwarno and Public Senior High School 1 Bayat. This school was chosen because it is located in an earthquake-prone area and was affected by the 2006 earthquake. The sample number includes 26 elementary school students, 173 junior high school students and 75 high school students. The sampling technique used is purposive sampling. Data collection uses questionnaires to obtain student knowledge and data on student needs, and technical data analysis is done using the *t*-test and Mann–Whitney test. The results of the study showed that (1) there was no difference in the knowledge of male and female students and (2) the needs of students in disaster learning included up-to-date disaster learning materials, adequate facilities and infrastructure, varied and fun learning methods and fun learning media. The findings provide critical insights for policymakers and educators to enhance disaster education programmes with a focus on inclusivity and student-centred approaches.

**Contribution:**

This study underscores the importance of designing disaster education tailored to students’ characteristics and needs, ensuring relevance and engagement. The learning strategies developed are more targeted by understanding children’s disaster characteristics and needs.

## Introduction

Disaster is one of the events that threatens human life. Disasters are unforeseen occurrences that result in significant human losses (Musiyam et al. 2019; Suwarno et al. 2025). One type of disaster that brings severe damage is earthquakes (Wang et al. 2020). An earthquake in an area, even for a few seconds, can cause severe damage and casualties (Baytiyeh 2015). Earthquakes are an inevitable calamity; however, the resultant losses can be mitigated by effective disaster management strategies (Qiu et al. 2024). Over the last few years, earthquakes have hit several regions in Indonesia, for example, the 2004 earthquake and tsunami in Aceh; the 2009 earthquake in Padang, West Sumatra; the 2018 earthquake in Palu, Central Sulawesi; the 2018 earthquake in Lombok, West Nusa Tenggara and the 2022 earthquake in Cianjur, West Java. The earthquake damaged infrastructure and caused loss of life, partly because of unpreparedness in dealing with earthquake disasters (Xia et al. 2021).

Earthquakes occur in disaster-prone areas, such as in plate subduction areas. Java Island is one of the islands in Indonesia located at the confluence of the Eurasian, Pacific and Indo-Australian Plates (Sayekti et al. 2024). All areas in the southern part of Java Island are prone to disaster events, and some areas are prone to earthquakes followed by tsunamis. This causes Java Island to be known as one of the islands with high vulnerability to earthquake disasters. It can be concluded that most of the population on the island of Java lives in areas prone to earthquakes. This is made worse by the fact that, based on Indonesian statistical data, population distribution is mainly concentrated on the island of Java.

**FIGURE 1:**
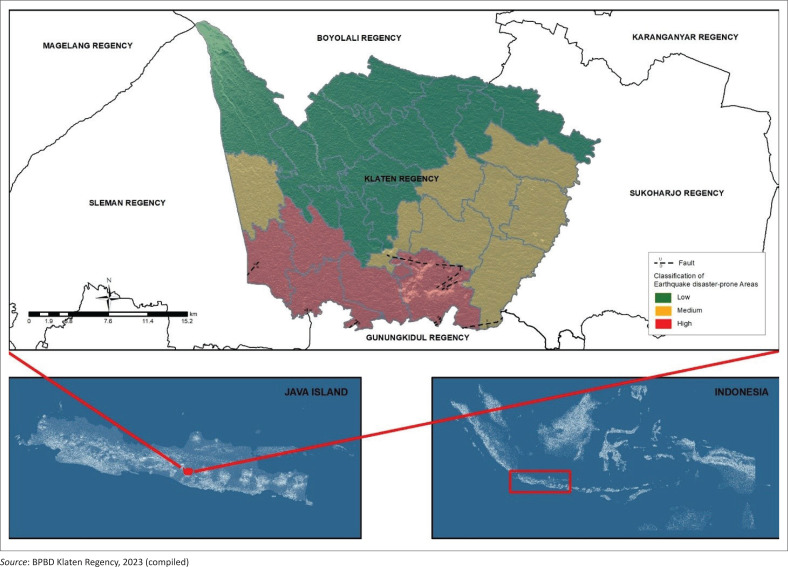
Earthquake disaster-prone area of Klaten Regency.

One of the areas on the island of Java that is prone to earthquakes is Klaten Regency. In Indonesia, a regency refers to an administrative region that is subordinate to a province and typically encompasses both urban and rural areas. The earthquake disaster that claimed lives and caused severe damage occurred in 2006. The earthquake in Klaten was exacerbated by the presence of the Opak Fault, one of the active faults in Klaten Regency. This resulted in areas traversed by the Opak Fault experiencing more severe damage compared to other regions. The earthquake’s epicentre came from the south of the Special Region of Yogyakarta, which had a magnitude of 6.4 on the moment magnitude scale. Based on data from the Regional Disaster Management Agency of Klaten Regency, the total number of victims was 1045 and around 95 892 buildings were destroyed. The severely affected sub-districts are near the Jiwo and Opak faults. This fault area is known to be easier to transmit earthquake vibrations. The sub-districts include Prambanan, Gantiwarno, Wedi and Bayat. The fault in Klaten Regency can be seen in Figure 1.

Disaster risk reduction follow-up must be carried out comprehensively from a global scale to a local scale (Renwick 2017). The Klaten Regency government issued a particular disaster management policy following the earthquake disaster. In addition, disaster safety education through schools was also pioneered, such as establishing ‘disaster-safe schools’, ‘disaster extracurriculars’ and disaster simulation programmes in schools. These efforts integrate disaster risk reduction through school education (Pfefferbaum, Pfefferbaum & Van Horn 2018). Education plays a role as a platform in efforts to reduce disaster risk (Park 2020). Integrating disaster education into the school curriculum is one of the strategies to improve disaster preparedness (Nurdin et al. 2017).

The current implementation of education has focused on the differences and needs of children, as stated in the education curriculum in Indonesia today. Children are not homogeneous but have different characteristics such as age, gender, beliefs, socio-economic conditions, culture and family structure (Amri et al. 2018). Furthermore, from the view of geography, men and women have differences in understanding phenomena in a place and space (Corotis & Enarson 2007). Such topics deserve further investigation (Trisnawati et al. 2025). Therefore, disaster risk reduction education needs to consider individual differences and needs so that its implementation can be optimal (Gaillard, Gorman-Murray & Fordham 2017). This study aims to determine the difference in school student knowledge and the difference in student needs in earthquake disaster learning based on gender. The hypothesis in the study was developed from the formulation of the first problem, namely that there is a difference in knowledge of earthquake disasters between male and female students in Klaten Regency, with male students demonstrating a higher level of earthquake-related knowledge compared to their female counterparts.

## Methodology

This study is quantitative research with a comparative design. The research was conducted at Public Junior High School Jabung, Public Junior High School 1 Bayat, Private Vocational High School Berbudi Gantiwarno and Public Senior High School 1 Bayat. These four schools were chosen because they are in areas prone to earthquake and have been affected by an earthquake in 2006. In Klaten Regency, there were 1045 fatalities and 18 127 injuries. Thousands of houses and public facilities were damaged, forcing hundreds of thousands of residents to flee, with the Bayat and Gantiwarno subdistricts suffering the most damage and casualties. The impact on schools was mostly physical damage to educational facilities; however, no fatalities were reported within the school setting. Nonetheless, the majority of students at the afflicted schools were from Bayat and Gantiwarno; therefore, they were directly affected by the 2006 earthquake disaster.

**FIGURE 2:**
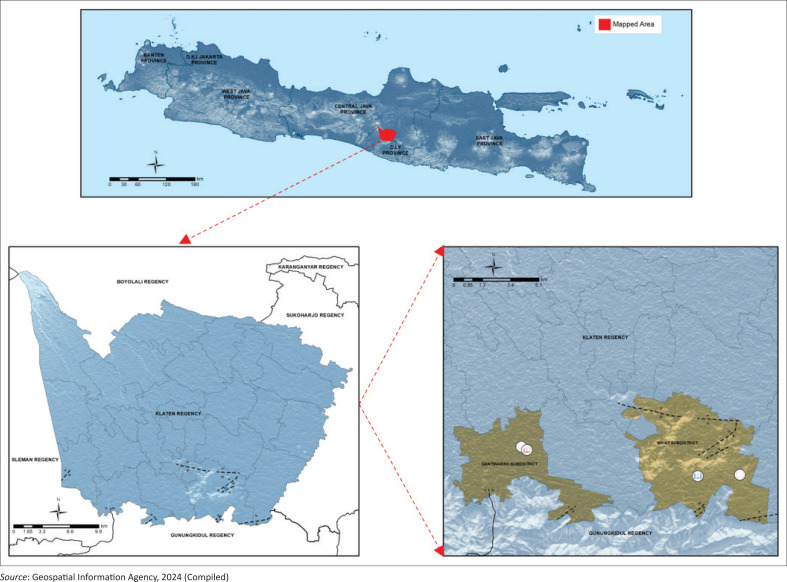
The study area.

The selection of this location is also based on the consideration that the school’s location is in a fault area in Klaten Regency. As previously known, the presence of faults causes the transmission of earthquake vibrations to be more significant. The location of the school against the fault can be seen in Figure 2. A dotted black line represents the fault. In Figure 2, it can be seen that the selected school is in the area where the fault passes. Purposive sampling was chosen to ensure participants represented schools in fault areas with historical earthquake impact. This method maximised relevance but may limit broader applicability. The sampling approach may not capture diverse perspectives from schools unaffected by earthquakes, potentially skewing findings. The considerations in this sampling are (1) students’ ability to fill out questions and questionnaires, (2) students’ activeness and communication skills and (3) recommendations from teachers regarding students who are most likely to fill out questions and questionnaires. Based on these considerations, a sample of 26 elementary school students, 173 junior high school students and 75 high school students was obtained. This is significant because most Indonesian school students live quite close to their schools as a result of the government’s implementation of a zoning regulation designed to provide fair access to education. This regulation requires students to attend schools in their home communities.

Data collection technique using questionnaires: The questionnaire items were adapted from the (Hidayati et al. 2006). Indicators of disaster knowledge questions include the definition of disasters, events that can cause disasters, causes of disasters, disaster impacts, characteristics of disasters, disaster mitigation, disaster information and local knowledge. The answer options are ‘yes’, ‘no’ and ‘I do not know’. Students’ knowledge was then classified into three categories, namely high (53–79), medium (27–53) and low (0–26). The maximum score is 79 if the students correctly respond all the questions.

Students’ needs regarding earthquake learning were measured using a questionnaire of 19 questions with indicators of disaster learning implementation, disaster materials, facilities and infrastructure, learning methods, learning media and resources and disaster learning assessments. The questionnaire used is mixed, meaning that the answer choices provided consist of two types: closed and open questions. The closed answer choice includes yes and no answer choices. Meanwhile, students can write answers to open questions according to their experiences. The genders in this study are male and female students.

Data analysis techniques include descriptive statistical presentation techniques and classical assumption tests in the form of normality and homogeneity tests. Test the hypothesis using the *t*-test (if the prerequisite test is met) and the Mann–Whitney test (if the prerequisite test is not met).

### Ethical considerations

Ethical clearance to conduct this study was obtained from Universitas Muhammadiyah Surakarta’s Faculty of Teacher Training and Education (No. 265/B.3-II/P.GEO/VIII/2023).

## Results

Students in a school tend to come close to the school because of the enactment of zoning policies in the admission of new students in Indonesia. New student admission zoning is a system of placing students in the school location closest to students in public schools. This means the student’s residence is close to the school. All schools in this study have been affected by the earthquake.

In this study, there are more female students (55%) than male students (45%). Most students have experienced an earthquake event (63%), but only 28% have obtained a disaster simulation. An overview of the respondents’ demographic profile is provided in Table 1.

**TABLE 1:** Demographic statistic of respondent.

Demographic statistics	Number	%
**Gender**		
Male	123	45
Female	151	55
**Education level**		
Elementary school	26	10
Junior high school	173	63
Senior high school	75	27
**Earthquake experience**		
Have	172	63
Do not have	102	37
**Disaster simulation experience**		
Have	76	28
Do not have	198	72

### Student knowledge of earthquake disasters in Klaten Regency

Table 2 describes the number of students who answered each question correctly. The more students answer correctly, the higher their knowledge.

**TABLE 2:** Students’ knowledge of earthquake disasters in Klaten Regency.

Questions	Students that responded correctly (%)					
	ES		JS		SS	
	M	F	M	F	M	F
**Definition of natural disasters**						
Natural events that interfere with human life	87	45	75	93	88	94
Human behaviour that causes damage to nature	33	9	28	27	16	76
Social or political unrest	60	55	63	76	76	4
Traffic accidents	73	73	65	72	64	4
**Events that can cause disasters**						
Earthquake	93	73	94	99	96	96
Tsunami	60	82	90	96	84	92
Flood	73	64	89	84	88	84
Landslide	87	73	94	88	92	90
Volcanic eruptions	80	55	94	97	88	98
Storm	47	64	92	89	96	86
**Causes of the disaster**						
Earth’s crustal shift	67	82	94	98	96	98
Erupting volcano	73	36	77	88	76	80
Landslide	60	18	42	37	32	56
Hurricanes and thunderstorms	67	55	65	66	76	8
Oil drilling	20	18	40	32	28	22
**Disaster impact**						
Tsunami	67	27	83	84	72	82
Landslide	87	82	87	78	76	86
Flood	53	45	30	19	24	22
Fire	20	9	12	9	4	4
Soil subduction	60	36	86	88	76	74
Erupting volcano	33	36	41	30	48	54
**Characteristics of the disaster**						
Is the day and hour of the earthquake known?	80	64	58	58	64	20
Earthquakes make people feel dizzy	47	36	41	47	44	50
Earthquakes cause strong and loud shaking so that people cannot stand	80	91	80	87	84	88
The tremors of the earthquake occurred for quite a long time and were followed by smaller aftershocks	47	73	78	83	76	80
Cracked or collapsed buildings	93	82	81	91	96	84
**Disaster mitigation**						
Take shelter under a sturdy table with a grip on the leg of the table	67	73	76	82	80	86
Stay away from bookshelves, items and hanging objects	73	91	77	92	92	88
Stay away from windows and glass walls	73	82	81	94	88	84
Get out of the room regularly (not jostling)	73	82	81	83	84	82
Running to the open space	40	45	87	94	76	74
**Disaster information**						
Earthquake disaster lessons	80	64	72	80	68	86
Disaster warning	53	36	63	70	52	64
First aid	73	55	71	79	72	78
Rescue and evacuation	53	36	65	73	60	74
Other	60	36	49	50	56	54
**Local knowledge**						
Any earthquake can cause a tsunami	27	55	64	77	64	10
*A Dengkeng fault* exists in Klaten Regency	53	18	22	11	16	10
*The Dengkeng Fault* influences the magnitude of earthquakes in Klaten Regency	47	27	25	29	20	34

ES, elementary student; JS, junior high school student, HS, senior high school student; M, male; F, female.

Table 2 shows the proportion of correct answers by male and female students at three educational levels. The analysis of respondents’ answers is presented as a distribution reflecting the extent of students’ knowledge about earthquakes. If a student answered ‘yes’, it is assumed that the student possesses knowledge regarding the item in question. However, for items related to the characteristics of earthquakes and disaster information, responses are not assessed based on correct and incorrect answers. The table illustrates both strength and weakness in disaster knowledge among these groups.

The male elementary school students possess substantial knowledge regarding ‘events that can cause disaster’ (73%), reflecting a fairly strong understanding of factors that cause disasters. In contrast, the lowest performance was in ‘local knowledge’ (42%), which indicates that indigenous knowledge has not been generally recognised. Female students scored highest in ‘disaster mitigation’ (75%), which could be related to their engagement with preparedness practices. However, they received the lowest score in ‘local knowledge’ (33%) revealing that contextual elements are consistently difficult for both sexes at this level.

Male students in junior high school exhibit a strong understanding of ‘event that can cause disaster’ (92%) similar to their female peers’ performance and emphasising a deep comprehension of the concept at this stage. On the other hand, ‘local knowledge’ scores the smallest percentages (37%), which was in line with the elementary school pattern. Likewise, female students scored the highest (92%), in the category of ‘events that can cause disaster’. Despite having performed marginally better than male students, their lowest score in ‘local knowledge’ (39%), nevertheless, shows a notable lack of understanding of traditional or local disaster-related information.

Male students in high school demonstrate considerable knowledge about ‘disaster mitigation’ (84%), showing an extensive comprehension of risk mitigation techniques. In addition, ‘local knowledge’ experienced the lowest score (33%), which confirmed a trend of inadequate performance in this category at all levels. Female students had the best score (71%) in ‘disaster information’, reflecting an adequate understanding of information sources and communication linked to disaster. However, they received the lowest score in ‘local knowledge’ (18%), which was the lowest result in all groups and categories and highlighted a crucial area for educational reform.

**FIGURE 3:**
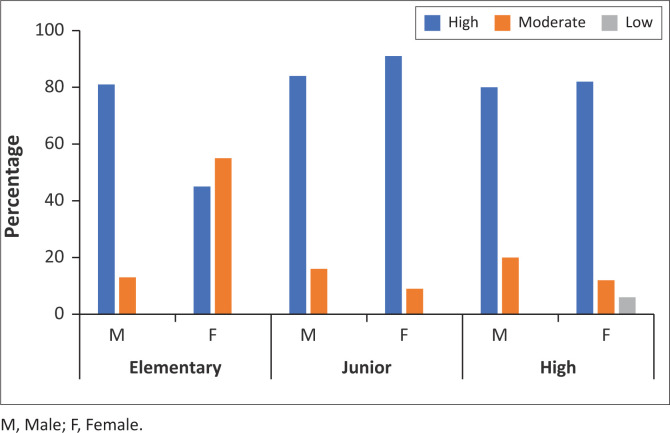
The percentages of students’ disaster knowledge across school level and gender.

Students consistently scored highest in the indicators ‘events that can cause disaster’ across all genders and educational levels, with both male and female students reaching a peak of 92% in junior secondary. Still across all groups, ‘local knowledge’ performed the worst, with female senior secondary students exhibiting the most noticeable deficiency (18%). A detailed comparison of students’ knowledge levels by educational stage and gender is presented in Figure 3.

### Statistical approach of knowledge differences between male and female students

At each educational level, to statistically calculate whether or not there is a difference in students’ knowledge, an analysis prerequisite test is carried out as a normality and homogeneity test of research data. The normality test was carried out using the Kolmogorov–Smirnov test. Meanwhile, the homogeneity test was carried out using the Levene test.

At the elementary level, male students have more correct answers to factors that can cause disaster, while female students answered more correctly on disaster mitigation indicators. Whereas in relation to ‘local knowledge’, male students frequently gave inaccurate responses, and female students performed similarly. An inferential test was carried out using prerequisite analysis of normality and homogeneity tests to determine the statistical difference. The finding of the normality test demonstrated that the data were normally distributed having a value for significance of 0.28 > 0.05. The data are homogeneously distributed, according to the homogeneity test result, which revealed a significance value of 0.553 > 0.05. Based on the two prerequisite tests, it can be stated that the criteria for the t-test analysis are accomplished.

It may be inferred from the *t*-test result that there is no difference in the knowledge of disasters between male and female students as the significant value was 0.96 > 0.05.

At the junior high school level, indicators of disaster-causing event were more accurately answered by male and female students. Both male and female students frequently provide incorrect answers when asked regarding local knowledge. Similarities exist among indicators at this level that could be addressed either less or more optimally. Based on the result of the normality test, it was possible to determine that the data were irregularly distributed because the normality value was 0.000 < 0.05. Additionally, it was possible to conclude that the data were homogeneous because the homogeneity test revealed that the signification value was 0.05 > 0.04. As the distribution is known to be aberrant and homogeneous according to the results of the analysis precondition test, the data fail to meet the requirements for the parametric statistical test. Therefore, the Mann–Whitney test is used as a non-parametric statistical test. Considering that the Mann–Whitney differential test yielded a significant value of 0.293 > 0.5, it can be stated that male and female students do not differ in their level of knowledge.

High-school male students performed better on the disaster mitigation indicator that female students did on the disaster information indicator. Both male and female students tend to give flawed responses when questioned about local knowledge. The data can be considered irregularly distributed as the normality test’s significance result is 0.000 < 0.05. The data are uniformly distributed, responding to the homogeneity test, which yielded a significance score of 0.258 > 0.05. The analysis criteria were still required to be fulfilled because the test finding proved that the data were homogeneous and abnormally distributed. Given the fact that the Mann–Whitney test result revealed a significance value of 0.290 > 0.05, it can be argued that male and female students have no differences in their understanding of disaster.

At all three levels, it is known that students lacked more local knowledge of disasters. Local knowledge is a type of knowledge that can strengthen individual capacity in local community-based disaster risk reduction (Kelman, Mercer & Gaillard 2012). Therefore, this study recommends integrating local knowledge materials into school disaster learning. Based on the results of statistical tests, it is concluded that the hypothesis in this study is rejected. The test results showed no significant difference in male and female students’ knowledge of earthquake disasters.

### Student needs in learning earthquake disaster

In this study, the needs of students are seen from two perspectives, namely, based on grade level and gender. The need in this disaster learning is based on the demand of students regarding disaster learning as determined by questionnaires and the various aspects of disaster learning implementation in schools that remain far from ideal (60%). Male and female students’ proportions of responses to how effectively disaster learning programmes have been implemented in schools are shown in Table 3. The current disaster learning programmes do not adequately address the needs and expectations of the students when the percentages drop below the predetermined level. The deficiency indicated an issue in the programme’s efficacy, pointing to areas that might require enhancements. Additionally, examining this gender-disaggregated data can reveal possible disparities in the programme’s perceptions by male and female students, which could guide more inclusive and focused intervention strategies. Eventually, meeting these unmet needs is essential to improving students’ preparedness and resilience in disaster-prone areas.

**TABLE 3:** Implementation of disaster education in schools.

No	Statement	Students’ responses (%)					
		ES		JS		SS	
		M	F	M	F	M	F
**Implementation**							
1	A disaster learning guide is used to study earthquake.	60	73	59	65	76	66
2	The school conveys the competencies, knowledge and skills related to earthquake that students must master.	60	45	64	74	76	66
3	Disaster learning activities can support students’ knowledge of earthquake.	80	91	86	97	92	96
4	Disaster learning activities can support students’ earthquake skills.	60	73	85	89	92	88
5	Disaster learning activities can support students’ attitudes towards earthquake.	93	73	86	93	76	100
**Disaster materials**							
6	The latest earthquake disaster subject matter (*up to date*) *is available.*	60	45	52	36	52	38
7	Students can easily understand the subject matter of earthquake.	80	64	79	85	80	88
8	Disaster subject matter can provide skills in dealing with earthquake.	93	82	88	95	96	98
**Facilities and infrastructure**							
9	There are sufficient disaster learning facilities.	80	45	60	58	48	52
10	There is sufficient earthquake disaster learning infrastructure.	67	36	59	63	56	44
11	The purpose of learning about earthquake disasters has been formulated.	93	73	72	73	68	62
**Method**							
12	The earthquake disaster learning methods used are varied and fun.	73	55	62	71	64	50
13	The earthquake disaster learning methods can activate students.	87	73	77	91	96	90
**Media and learning resources**							
14	Appropriate earthquake disaster learning materials and books are available.	53	73	56	47	80	60
15	There are appropriate and fun earthquake disaster learning media.	67	73	59	62	64	62
16	ICT or computer-based learning media are available.	40	27	67	71	64	68
**Evaluation**							
17	Appropriate assessment instruments are available.	73	73	70	70	76	76
18	The learning process develops assessment instruments.	73	73	77	85	80	84
19	Assessment techniques are in accordance with the learning process.	87	82	79	88	84	88

ES, elementary student; JS, junior high school student, HS, senior high school student; M, male; F, female; ICT, Information and Communication Technology.

**FIGURE 4:**
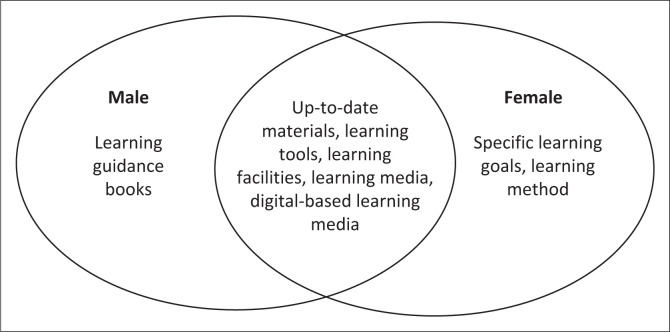
Students’ needs for disaster learning based on gender.

The implementation of disaster risk reduction education in schools has several strengths, as shown in Table 3. These include the programme’s support for disaster knowledge improvement (90%), the presence of activities to enhance knowledge, skills and understanding of earthquake disaster (85%), the inclusion of a disaster subject in the curriculum (81%) and the programme’s encouragement of students to participate actively in the learning process. Meanwhile, challenges in the implementation of disaster risk reduction education in schools include an updated disaster material (51%), inadequate defence facilities and infrastructure (63%), less varied and less fun learning methods (94%), the absence of disaster learning support books (66%) and the absence of Information and Communication Technology (ICT)-based learning media (61%). The needs of students in disaster learning are presented in Figure 4.

Male and female students have both distinct and comparable needs in several aspects. While male students have a need for learning guidance books, female students demonstrate a need for specific learning goals and learning methods. Furthermore, a number of comparable needs include up-to-date materials, learning tools, learning facilities, learning media and digital-based learning media.

**FIGURE 5:**
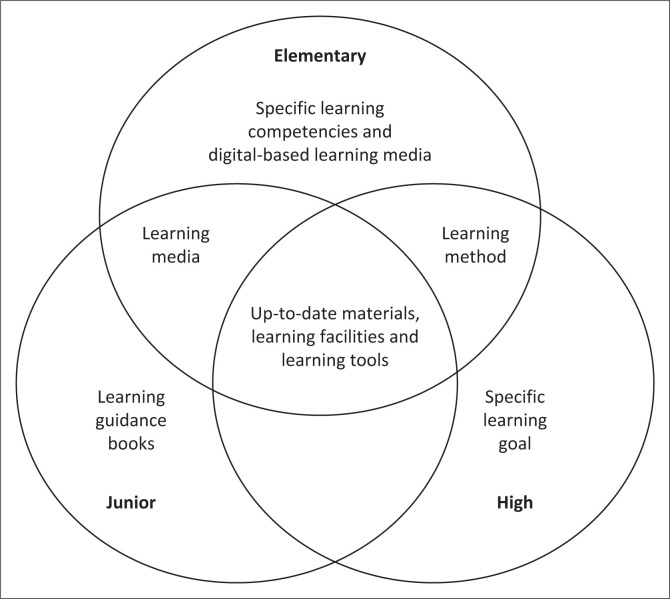
Students’ need for disaster learning based on school level.

Figure 5 illustrates the needs for students at each level. Specific learning competencies and digital-based learning media are required at elementary school, along with learning guidance book for junior high school. Specific learning goal must be developed at the high school level. Furthermore, elementary and senior high school students emphasise learning methods, while elementary and junior high school share a common need, namely learning media. Up-to-date materials, learning facilities and learning tools are needed to support learning at each level.

Students’ needs for disaster learning programmes are similar and different at each educational level. Developmental stages, cognitive capacities and the unique environments that learners at various ages encounter all have an impact on these variances. Although the fundamentals of disaster preparedness may be globally significant, the strategies for the successful achievement of this programme must be adjusted to each educational stage. Designing an effective disaster learning programme that is both relevant and interesting for all students requires an understanding of these subtle variances.

## Discussion

Knowledge is one of the essential competencies that every individual must have in order to be able to make the right decisions in various areas of life (Tuladhar et al. 2015). Disaster knowledge is urgent for students living in disaster-prone areas because it can be used as one of the competencies in reducing disaster risk for themselves (Mutch & Gawith 2014). Every individual living in a disaster area must improve his or her competence in dealing with disasters by increasing knowledge and awareness of disasters (Barua et al. 2020).

Children are one of the parts of society that are vulnerable to earthquake disasters; therefore, disaster risk reduction education for children is critical to be provided (Islam 2021). There are several minimum indicators that students must master to have good knowledge, including knowing what a disaster is, the causes of disasters, the impact of disasters, the characteristics of disasters, disaster mitigation, disaster information and local knowledge of the area. Knowledge of these aspects can help deal with disaster emergencies, ultimately reducing the fatal impact of disaster events (Musacchio et al. 2016). It can be interpreted that disaster knowledge can improve disaster preparedness (Gülsoy et al. 2024).

Schools must implement disaster risk reduction education (Shiwaku et al. 2016). Disaster education in schools is one of the initial stages of increasing knowledge about disasters (Gouramanis & Morales Ramirez 2021). In addition, through disaster education in schools, students can actively contribute to disaster prevention and risk reduction (Tuladhar et al. 2014). Disaster risk reduction education programmes have been implemented at every level of education, from elementary to junior high to high school. Each level has a different programme. At the elementary level, disaster recognition is integrated into science subjects; at the junior high school level, it is integrated into integrated social studies subjects, and at the high school level, it is integrated into geography subjects. In addition to extracurricular programmes, disaster integration is also carried out in extracurricular programmes. Klaten Regency has ‘disaster mitigation extracurriculars’, ‘cheerful, peaceful, and disaster preparedness (CERDAS) schools’, ‘river schools’ and ‘disaster learning outbound’ (Sunarhadi et al. 2018). Additionally, ‘The Youth Red Cross and Scout’ support the programme. These programmes aim to improve students’ disaster preparedness (Dwiningrum 2017).

Although there have been many disaster education programmes in schools, this study determined that students’ knowledge need to be improved in number of areas, including ‘local knowledge’. This can be caused by the need for optimal integration of disaster materials in the curriculum, which leads to the suboptimal success of disaster education in schools (Kitagawa 2015). This is possible because the portion of disaster materials at each level of education is different. Most disaster materials are at the high school level, while the least is at the elementary level. Disaster material is implied in one specific chapter, like at the elementary and junior high school levels. Disaster materials only appear in social studies subjects. In addition, students’ disaster knowledge has not been optimal because the integration of disaster reduction programmes through extracurricular activities has not been optimal.

Elementary school students possess basic knowledge of the ‘definition of natural disaster’, the various events that can lead to disasters and the fact that earthquakes may trigger landslides. They also recognise that earthquake occur unexpectedly at any time. However, only limited number of students are aware that, during an earthquake, it is imperative to evacuate promptly to an open area for safety. Additionally, their understanding of local hazard contexts and specific seismic characteristics remains insufficient. This gap highlights the need for more comprehensive and contextually relevant disaster education at elementary level. This issue is possible because students have never conducted earthquake disaster mitigation simulations before. In addition, even if knowledge is crucial, developing practical skill is essential to increasing disaster risk reduction capacity to higher level (Gouramanis & Morales Ramirez 2021).

Both male and female junior high school students exhibit a strong grasp of the ‘event that can cause disaster’, including an awareness of earthquakes brought on by tectonic plate movements. They also have an extensive understanding of disaster mitigation followed by good awareness of the possible effects of earthquakes, such as the triggering of landslides and tsunamis. However, more is needed to know whether earthquakes can be predicted and the continued impact of disaster events. Junior high school students have yet to experience the continued impact of earthquakes, such as landslides, fires and tsunamis. As far as students are concerned, the disasters that occur only cause damage to buildings and other infrastructure but do not cause other further disasters.

High-school students have good knowledge regarding the nature of earthquake disasters, the phenomena that cause earthquakes, some characteristics of earthquakes, disaster mitigation and the acquisition of earthquake disaster information. However, there is limited knowledge regarding the cause of disaster and impact of the earthquake, particularly among senior high school students.

At all levels, ‘local knowledge’ is still severely lacking for both male and female students. This is particularly important as disaster characteristics must be tailored to local circumstances, such as the existence of geological faults that might worsen the effects of earthquakes. This lack of ‘local knowledge’ is acceptable because of several factors. First of all, the earthquake that occurred in Klaten Regency was an unexpected event, and most of the locals did not anticipate it. Second of all, although the existence of Dengkeng Fault in Klaten Regency is theoretically acknowledged, little information about it has been widely disseminated, particularly in educational system. The limited inclusion of geological content in school curricula, along with other constraints, contributes to general public’s lack of awareness to local geological risk.

The results of the statistical test showed that there was no significant difference in knowledge of earthquake disasters between male and female students. This means that gender does not influence students’ knowledge about disasters. This is different from Chen and Lee (2012) and Choe, Kim and Ri (2020), where the results of the study found that there was an influence of gender on disaster knowledge. In this study, it is possible that other attributes of students can influence it, such as the presence or absence of disaster experience and whether or not they have obtained disaster mitigation education.

Students’ needs in disaster learning include clear learning objectives, updating disaster materials, learning facilities and infrastructure and disaster learning media. Children need fun learning tools in disaster-related learning (Ebbeck, Yim & Wei 2020). This reinforces that disaster learning needs to be fun and involve students’ active participation (Martin 2010).

Men and women have different characteristics when dealing with disasters, so it is essential to associate disaster studies with gender (Gaillard et al. 2017). Some studies say women are more vulnerable to disasters than men (Juran & Trivedi 2015). Therefore, in disaster management, it is essential to provide appropriate treatment for men and women (Saito 2012). The study results show that female students’ needs in disaster learning are more comprehensive than male students. The aspects developed come from materials, tools, guidance, learning methods, facilities and infrastructure to learning media. This is because of differences in response to disasters between male and female students (Chetry 2022). Apart from disasters, male and female students have different responses during learning (Akande 1999). Female students’ involvement is more emotional than male students (Bru et al. 2021). Because of this characteristic, in the disaster-strengthening programme, women must be prioritised (McNamara, Clissold & Westoby 2021).

## Conclusion

The study results show that students’ knowledge of earthquake disasters needs to be more comprehensive. It is still necessary to increase student knowledge to improve student disaster competencies. To enhance student disaster preparedness, educators should integrate comprehensive, up-to-date disaster content into the curriculum and conduct regular simulations tailored to student needs. Both male and female students have the same knowledge, so it can be concluded that gender is not a factor that affects students’ knowledge. Students’ needs for disaster learning include developing up-to-date disaster materials, learning facilities, specific learning goals, suitable learning methods, learning guidance, digital-based learning media and learning tools. In this study, it was also found that the needs of female students are more than those of male students. For the development of disaster learning programmes, it is essential to pay attention to the needs of students. It is also essential to look at the difference in needs between male and female students because there are more needs for female students than for male students. The findings support the need for Disaster Risk Reducation (DRR) education policies that emphasise student-centric and gender-responsive approaches.
